# Early psycho-social support of pregnant women at risk for preeclampsia and fetal intrauterine growth restriction

**DOI:** 10.1186/s12884-025-08363-9

**Published:** 2025-10-27

**Authors:** Franziska Epple, Jörg Reichert, Mario Rüdiger, Cahit Birdir, Lars Mense

**Affiliations:** 1 Department of Pediatrics, Division of Neonatology & Pediatric Intensive Care Medicine, University Hospital Carl Gustav Carus Dresden, TU Dresden, German Center for Child and Adolescent Health (DZKJ), Partner Site Leipzig/Dresden, Fetscherstrasse 74, 01307 Dresden, Germany; 2 Department of Gynecology, Division of Obstetrics, University Hospital Carl Gustav Carus Dresden, TU Dresden, Fetscherstrasse 74, 01307 Dresden, Germany; 3 Saxony Center for Feto/Neonatal Health, TU Dresden, German Center for Child and Adolescent Health (DZKJ), Partner Site Leipzig/Dresden, Fetscherstrasse 74, 01307 Dresden, Germany

**Keywords:** Psychology, Social support, Social work, Risk assessment, IUGR, Mental health, High risk pregnancy, Maternal-fetal medicine

## Abstract

**Background:**

The risk of preeclampsia and intrauterine growth restriction can be assessed in the first trimester combining maternal characteristics, biophysical and biochemical measurements. The early risk identification allows targeted follow up and prophylactic treatment with acetylsalicylic acid but potentially influences the pregnant woman’s experience and mental well-being. A structured follow-up involving psycho-social support might improve patient care and decrease the burden of being identified as a woman at risk of a potentially life-threatening pregnancy complication. We describe the utilization of an early psycho-social intervention and concerns raised by the participants.

**Methods:**

Secondary analysis of the early psycho-social interventions as part of a prospective cohort study.

**Results:**

92.1% (441/479) participated in the early psycho-social intervention. 86.6% had at least one risk and load factor: 56.7% reported fears, 44.0% had mental illnesses in past or present and 36.7% reported social constraints. Experiences of violence were reported by 25.9%. Additional support was required in 27.7% of cases. More than 90% of women recommended a similar conversation for other women in a similar situation.

**Conclusions:**

An early psycho-social intervention for pregnant women at risk is well accepted and many report risk and load factors which can be addressed by the psychologist. Some women require additional support which can be instituted afterwards.

**Trial registration:**

The study was registered at clinicaltrials.gov (number NCT04514276, registered 14/08/2020) and DRKS (number DRKS00017713, registered 09/06/2020).

## Background

Preeclampsia (PE) and intrauterine growth restriction (IUGR) are well described pregnancy complications which are potentially life-threatening for mother and fetus. Both diseases originate from placental dysfunction with PE being a maternal hypertensive disorder in pregnancy [1] while IUGR is the failure of the fetus to achieve its genetically determined growth potential [2]. Nowadays, an early risk assessment is feasible using a combination of maternal characteristics, biophysical and biochemical measurements at 11 + 0 to 13 + 6 weeks of gestational age following the Fetal Medicine Foundation’s protocol [3]. Using this strategy, 76% of women later developing preterm PE and 38% of women with term PE were identified in a previous study [4]. The ASPRE trial demonstrated that the treatment of women at risk (identified using the early risk assessment strategy mentioned previously) with low dose acetylsalicylic acid (aspirin) decreased the incidence of preterm preeclampsia [5] and small-for-gestational age newborns (SGA) [6]. Importantly, aspirin treatment starting after 16 weeks of gestational age is ineffective in reducing PE and IUGR [7], thus identification of pregnant women at risk early in pregnancy is crucial although fears might be caused this early in pregnancy.

The early identification as a pregnancy ‘at risk’ potentially influences the pregnant woman’s experience and mental well-being [8]. In the ASPRE trial, a subset of women received a longitudinal psychological assessment: Women at high PE-risk reported more depressive symptoms than women at low PE-risk in the second but not the third trimester [9]. Simeone et al. found no increase in anxiety among women undergoing early PE screening [10]. Surprisingly, no differences were seen between women identified as low or high risk. In a small qualitative study, early PE screening was generally well perceived among women of high and low risk groups but some women at high risk reported their pregnancy being a source of worry after PE screening [11]. Since antenatal anxiety and depression influence maternal morbidity and infant outcome [12, 13], diagnosis and treatment are important. The importance of effective stress-coping strategies is well described among various health issues [14, 15].Unfortunately, supportive interventions such as counselling by a psychologist or social worker were not evaluated in the aforementioned trials. Thus, the problem was identified but not the effectiveness of treatment options.

Psychosocial support programs demonstrated their effectiveness in reducing stress of patients and parents in other circumstances, such as acute pediatric hospitalization [16], pediatric cancer [17] and perinatal loss [18]. After pediatric death, bereavement counselling was more effective in reducing grief than peer support [18]. Interestingly, stress reduction by group-based psychoeducation was effective in reducing the percentage of SGA newborns compared to routine antenatal care [19]. It is well described, that coping strategies influence stress perception [20]. Among hospitalized high-risk pregnant women, mindfulness-based interventions were effective in reducing anxiety [21] and problem-focused coping strategies were associated with less anxiety, depression and stress [22].Therefore, it seems important to offer early psychosocial support to women undergoing early PE screening or being diagnosed with other risks for mother or fetus in pregnancy.

The healthcare research project *FetoNeoPfad* (feto-neonatal pathway), funded by the Innovationsfonds of the Federal Joint Committee (Gemeinsamer Bundesausschuss), addressed the need for multidisciplinary care of women at risk of PE and fetal IUGR to improve the maternal and neonatal outcome in the model region [23]. Community obstetricians, maternal-fetal medicine specialists, neonatologists, pediatricians, psychologists and nurses cooperated in the project to offer a clearly structured, integrated care from the beginning of the pregnancy until one year after delivery. Interdisciplinary discussions of patient cases optimized the integrated care. Psychosocial support at pre-defined time points throughout pregnancy and the first year after delivery was implemented as part of the multidisciplinary care. The interventions focused on different aspects of psychosocial care, mainly information and relationship building, giving the perception of control, coping with stress and assessing the newborn’s and its parent’s regulation. This study evaluates the utilization of the first, early contact between psychologist and the pregnant woman. This contact was scheduled after risk identification between 10 and 16 weeks pregnancy. We report the psycho-social risk and load factors and the perspectives of patients and provider regarding the intervention.

## Methods

The *FetoNeoPfad* is a research project incorporating various interventions to improve the quality of care among pregnant women at risk for PE or IUGR.

### Patient management in the *FetoNeoPfad*

Pregnant women living in two regions of Germany (Eastern Saxony and Eastern Thuringia) were enrolled into the research project by their community gynecologists if risk factors for PE or fetal IUGR were present by maternal history (maternal age above 35 years, history of PE or IUGR, chronic arterial hypertension, diabetes mellitus and others). A propensity score-matched control group was formed from pregnant women in two other regions of Germany (remaining Saxony and Thuringia, identified by postal codes). A PE screening between 11 + 0 and 13 + 6 weeks of gestational age was performed by specialized obstetricians, certified by the Fetal Medicine Foundation, London and according to their guidelines [3]. Aspirin prophylaxis was suggested as per the criteria of the ASPRE study and women were followed by specialized obstetricians. Patient cases were discussed among all care providers at the perinatal centers throughout pregnancy, involving obstetricians, neonatologists and psychologists. Thereby, psychosocial issues could be incorporated into patient care at any time during out- and inpatient stay, including, but not limited to, additional psychosocial support, appointments with physicians or lactation consultants etc. Delivery was planned according to risk profile and parental preferences. Newborns requiring neonatal intensive care were followed in the *FetoNeoPfad* during hospital stay and until one year of age by their local pediatricians. More in-depth information of this research project have been published before [23].

### Psychosocial support

Psychosocial support was planned at five pre-defined time points from the first trimester until the end of the first year of the infant’s life. The time points were chosen to support the family at crucial events during pregnancy and postpartum period (risk identification, diagnosis of IUGR or PE, potential preterm delivery, after discharge of their child, after the first year of life). Follow-up appointments were possible as needed. The five pre-defined appointments were focused on different topics (Table 1). While Module 3 had been implemented and evaluated in routine care at our perinatal center before, the other modules have been newly developed. Conversations were held in person or by telephone to reduce the burden for outpatient visits during pregnancy. Modules 2–5 were offered to women after diagnosis of IUGR or PE only.

**Table 1:** Time points and topics of psycho-social support

**Module**	**Module description**
Module 1: Information	*Risk identification (10–16 weeks)* Telephone call: Information, relationship building, potential burdens, psychoeducation (normalizing stress reaction, coping with fears), compliance, history taking (KINDEX[24]), resource activation, organizing further psycho-social support as needed
Module 2: Advice	*Diagnosis of IUGR or PE (24–30 weeks)* Telephone call or visit on the ward: potential burdens, resource activation; information regarding preterm birth, visiting the neonatal intensive care unit as needed, organizing further psycho-social support as needed
Module 3: Accompaniment	*After admission at the perinatal center for potential delivery*: Visits on the ward: Attachment oriented/supportive advice, crisis intervention (empowerment, forward-looking approach), theoretical and practical guidance in preterm newborn care, organizing further psycho-social support as needed [25]
Module 4: Prevention	*Four weeks after discharge*: Screening questionnaire: Neonatal regulatory disorders, maternal psychological issues, experienced stress; follow-up telephone call to review the findings, organizing developmental psychological support as needed
Module 5: Support	*One year of age*: Screening questionnaire: Neonatal regulatory disorders, maternal psychological issues, experienced stress; standardized assessment of infant development

For module 1, all women included into the research project were proactively contacted by a research administrator and a telephone call from the study psychologist appointed. Psychosocial risk factors (including present fears, mental illnesses, social constraints, problems in partnership, experiences of violence and consumption of alcohol or nicotine) [26] were assessed following a predefined interview guide. Social constraints were deemed relevant if the assessor or the patient felt effects on the patient’s life’s conditions during pregnancy and potentially after delivery [27]. Caution was paid to ensure a trustful patient-provider relationship. The primary intent of the call was to assess individual needs and to develop potential solutions. After the identification of psychosocial risk factors, self-efficacy and individual resources were assessed. If self-efficacy or resources were lacking, additional resources were provided, including, but not limited to counselling on protection against violence or addiction, financial support agencies, psychotherapy or other problem-specific support programs. A lack of self-efficacy was diagnosed in women who were not able to articulate coping strategies or plans how to deal with these risk factors. At the end of the conversation, the women were asked for their experience with the conversation.

The study psychologist (F.E., “provider”) has been trained in clinical psychology, trauma-informed care, crisis intervention, care after domestic violence and has had extensive experience in counselling women in pregnancy and during stay of their offspring in neonatology.

### Data collection and statistics

Data from the consultations of module 1 which was typically held between 11 and 15 weeks of pregnancy are analysed. Data from other modules was not included since the early intervention after risk assessment should be evaluated. Included were all conversations held with participants in Eastern Saxony. Sample size calculation was performed for the primary outcome of the *FetoNeoPfad* (number of stillbirths and postnatal transfers into neonatal intensive care units). Data was collected using REDCap electronic data capture tools hosted at TU Dresden [28, 29] and analysed using SPSS 28 (IBM, New York, USA). Data was validated by LM and missing data was not imputed. Data is presented as median (25th percentile; 75th percentile) and minimum, maximum due to non-normal distribution.

## Results

### Description of the study cohort

492 women were eligible for Module 1 psycho-social consultations in the years of 2020 till 2022. Only 8 women (1.6%) declined the offer. Asked for reasons to decline, time constraints or no needs were stated by the participants. Research administrators were unsuccessful to contact 26 women (5.4%) per telephone or mail. Psycho-social consultations were offered in German or English and held by telephone; language barriers impeded conversations in 13 women (2.6%) and could not be circumvented by interpreters. Thereby, utilization rate was 92.1% (441/479).

The median age of the women was 33.6 (30.2; 36.9) years (min: 18; max: 49). The median length of the conversations was 47 (37; 59) min (min: 10, max. 95). Median time point was 11 (7; 20) days after enrollment in the research project. Although the conversations were planned at the earliest, time constraints or difficulties contacting the participants were barriers and caused a wide time span for the first consultation (min: 1 day, max: 153 days).

### Risk factors

**Fig. 1:**
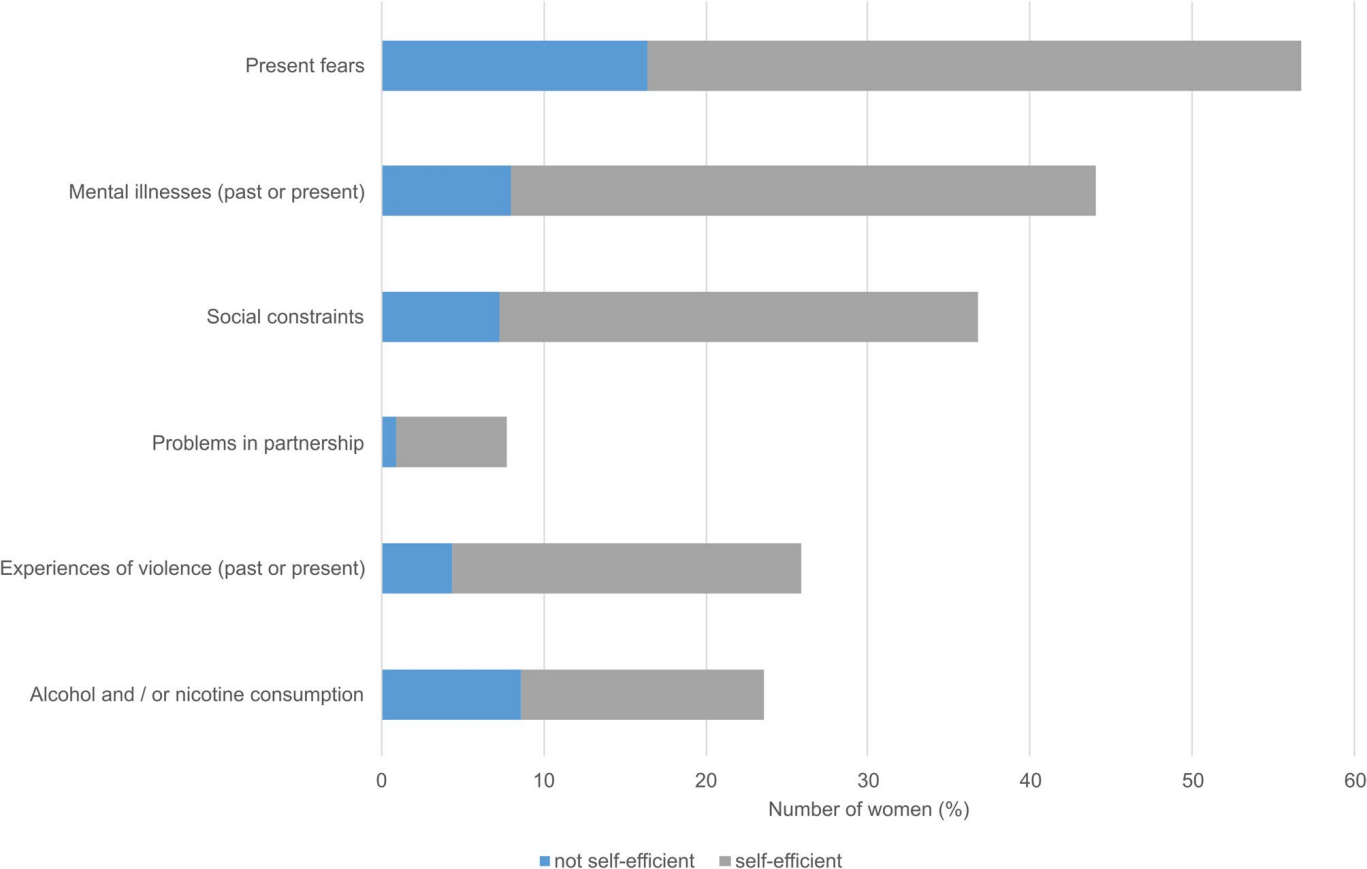
Psycho-social risk factors and maternal self-efficacy. Percentage of women reporting psycho-social risk factors, divided by self-efficient handling as assessed by the study psychologist

During the psychological consultations, the women were asked about their experience with their pregnancy. They were asked to recall how they perceived the diagnosis of being at risk for PE or IUGR. Oftentimes, the women asked to discuss the result of their risk calculation again and to interpret the risk ratio which seemed quite abstract for many women. The psychologist provided information on PE and IUGR in the sense of an educational conversation if asked for by the women. Psycho-social risk and load factors were cautiously assessed (Fig. 1). 382/441 of the women reported at least one psycho-social risk factor (86.6%), 242/441 (54.9%) more than one. 250/441 of the women (56.7%) reported present fears. In 72/250 (28.8%), self-efficacy seemed lacking and further resources were provided. 194/441 (44.0%) of the women reported mental illnesses in their past or present. 35/194 (18.0%) demonstrated symptoms at presence without adequate medical or psychotherapeutic treatments.

Approximately one third of the women (162/441, 36.7%) reported social constraints: These involved financial concerns, difficulties finding appropriately sized housing, issues with pregnancy-associated bureaucracy and, in some cases, homelessness or an overall desolate living situation. 32/162 (19.8%) lacked self-efficacy regarding their social constraints. 34/441 (7.7%) talked about problems in their partnership, lacking self-efficacy in 4/34 (11.8%). 114/441 (25.9%) reported experiences of violence at some point of their life, including violence caused by their parents, through single occasions with strangers or in their social proximity. Rarely, violence in their actual partnership was reported. The majority had found efficient coping strategies, but 19/114 (16.7%) still suffered from their experiences of violence without self-efficient handling.

### Experiences gained from the psycho-social support conversations – the provider’s perspective

The phone calls were proactively scheduled by the research administrators and the offer was well perceived by the participants. The women described the telephone conversations as being pleasant. Using the telephone, long distance travel was avoided and the women were able to speak in their familiar surroundings. The provider had the impression that the contact was established very well during the first conversation in most cases. Additional support was deemed necessary in 27.7% of the cases (100/441) and women were brought into contact with appropriate support agencies. In most cases, women were referred to counseling centers for pregnant women which can convey financial support and offer advice for governmental support in Germany. Psychotherapy was required in some cases to work on traumatic birth experiences or severe anxiety. Three women were referred to a specialised counseling center for domestic violence. If women had not seized smoking, an online platform supported by the Federal Center for Health Education (BZgA) was recommended (www.iris-plattform.de).

The early contact to the women in Module 1 (at 10–16 weeks of pregnancy) proved helpful from the provider’s perspective if complications such as stillbirth or extreme prematurity occurred later in the pregnancy since a trustful patient-provider-relationship was already established. Some situations potentially causing child endangerment could be anticipated; theses included drug addictions, parental psychiatric diseases or educational violence among their offspring. Some women could be motivated to contact Early Childhood Interventions during pregnancy which is a publicly funded German-wide initiative for families in difficult situations and can promote family midwifes and other support as an additional resource for the families (https://www.fruehehilfen.de).

Since breast milk feeding is the optimal nutrition for all newborns including those being preterm or IUGR, previous breastfeeding experiences were evaluated in the first conversation. Negative experiences or reservations against breastfeeding were carefully addressed and a board-certified lactation consultant could be contacted for an antenatal conversation with the pregnant woman.

### Experiences with early psycho-social support conversations – the patient’s perspective

**Fig. 2:**
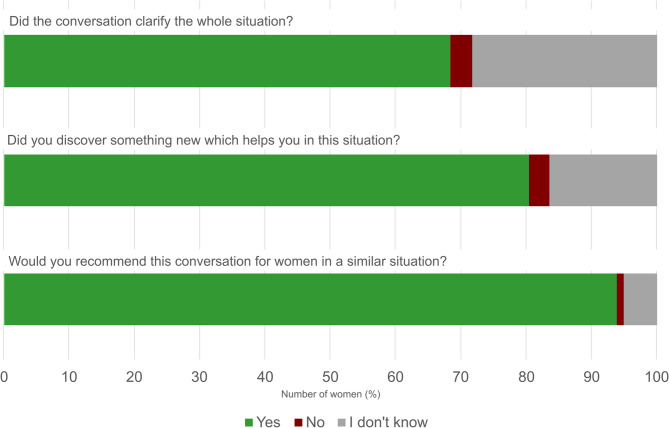
Self-reported patient’s experience. Self-reported evaluation of the mother’s perspective regarding the psycho-social conversation and their recommendation for other women in a similar situation

At the end of every encounter, the psychologist asked the woman about her experience (Fig. 2). The majority of women felt that they had discovered something new which helped them to cope with the new situation being diagnosed with a pregnancy at risk. Many felt that the conversation clarified the situation. Over 90% of the women recommended a conversation with a psychologist for women in a similar situation.

## Discussion

In this study, an early psycho-social intervention is provided to pregnant women after being identified at increased risk of PE and IUGR. An early psycho-social intervention has not been part of routine care or has been investigated in clinical research in Germany previously. We demonstrate a high utilization rate of 92% and a positive self-reported evaluation. The vast majority of patients would recommend a similar conversation for other pregnant women at risk. Psychosocial issues were present in many patients but most seemed to be able to manage them on their own. One out of four pregnant women received additional support.

### Identification as a pregnancy at risk causes stress

The antenatal care of pregnant women is aimed at ensuring maternal and fetal health during pregnancy. Recent developments enabled obstetricians not only to identify congenital anomalies but also to identify women at high risk for complications such as PE and IUGR [30]. This knowledge allows preventive care and close observation throughout the pregnancy but potentially increases the perceived stress of the pregnant woman. The psychological effects of PE screening are likely not fully comparable to other first trimester screening methods such as non-invasive prenatal testing (NIPT) or the nuchal translucency scan: Risk identification by the latter leads to subsequent investigations such as genetic testing and a definitive diagnosis in a timely manner. The uncertainty can be resolved although the diagnosis might have severe implications. Since PE can develop at any time during pregnancy, the risk identified by PE screening affects the pregnancy at length and no test can confirm or exclude PE ahead of its development.

In a subgroup of the ASPRE trial participants, women at high risk of PE reported more symptoms of depression in the second but not the third trimester, thus closer to the risk assessment [9]. Women at high risk changed their lifestyle if they had low depression scores. Those with high depression scores did not demonstrate lifestyle changes, indicating that health literacy is affected by psychological wellbeing.

Contrary, anxiety levels were similar among low and high risk women after PE screening in a previous trial [10] which assessed the participants within 1 h of diagnosis. In the ASPRE trial [9], the assessment was several weeks after initial counseling, thus coping with increased risks in pregnancy might be fluctuant over time and repeated assessments of pregnant women seem important. Both studies need to be cautiously interpreted since potential confounders were not assessed in-depth. In our study, the rate of psycho-social risk factors was surprisingly high but can hardly be compared to previous studies since a wider range of risks was evaluated [9, 10]. The length of time between risk identification and first psychosocial conversation was below three weeks in most patients of our cohort but some conversations could not be scheduled until much later. This delay potentially influences the mental well-being and the concerns reported. Unfortunately, our study was not designed to identify an optimal time-frame.

### Antenatal stress has neurocognitive consequences for the fetus and newborn

Maternal stress in pregnancy influences the neurodevelopment of the newborn by different pathways [31]. Epigenomic association studies recently demonstrated that stress in pregnancy is associated with changes in DNA methylation, involving areas of neuronal, immune and endocrine homeostasis [32]. Dysregulation of the hypothalamic-pituitary-adrenal axis is a common feature in the offspring after maternal stress in pregnancy [33] with potentially long-lasting effects [34]. The postnatal environment seems to influence the infant’s resilience and its development after antenatal stress exposure [35]. Therefore, adequate antenatal support for high risk pregnancies seem important from both a maternal and feto-neonatal perspective.

### Antenatal psycho-social interventions in high-risk pregnancies

Psychological interventions in high-risk pregnancies are neither implemented in routine care nor made easily accessible in Germany although a recent German guideline recommends antenatal psychosocial support for families expecting a preterm or sick newborn [36]. Limitations exist on multiple levels including assessment and awareness, access to care, individual experiences and have been described before, including other countries [37, 38]. The scientific evidence evaluating the effects of antenatal psychological interventions is scarce [39]. Contrary, postnatal support by a psychosocial team has been well studied and is shown effective in reducing parenting stress [25, 40], symptoms of traumatization [41] after preterm birth and improving cost-effectiveness [42]. Our data supports the implementation of antenatal psychosocial support for women with high risk pregnancies. It is noteworthy, that a significant number of women reported social constraints. We believe that antenatal support personnel should have the capacity to offer psychological as well as social assistance. The proactive contact from a research administrator to book an appointment helped to reach a utilization rate of over 90% which is high for a psychosocial invitation compared to other diseases [43].

### Strengths and limitations

Our study is one of the first evaluating early routine psycho-social support for pregnant women at risk of pregnancy complications. The high utilization rate ensures an unbiased view onto this risk group. Only a small proportion could not participate due to language barriers. The use of interpreters might lower that number but would complicate the conversation by phone significantly. Our data is based on individual observations instead of standardized psychological instruments. The psycho-social consultations were primarily focussed on patient care; thus the use of standardized questionnaires was waived. Thus, we present real-life data but external validity and reproducibility are limited. Due to the study design, no psychological data could be obtained from the control group.

Since the follow-up period was limited, we cannot evaluate long-term psychological consequences of early PE risk screening which should be considered for future trials. Although our study demonstrates that many women at risk of PE and/or IUGR report psycho-social risk factors and the early intervention is well perceived, the efficacy of the intervention cannot be evaluated since a control group and baseline data before risk identification is missing which was due to the study design. Further studies are required to demonstrate the efficacy and cost-effectiveness. Nevertheless, an implementation beyond PE and IUGR should be considered and will be evaluated as part of the research project “SafeBirth network” in Eastern Saxony [44, 45]. For the future, it is worth investigating how the pregnant woman’s partner is affected by the risk identification and how antenatal interventions are utilized by the partner.

## Conclusions

Pregnant women at increased risk to develop PE and/or IUGR agreed to participate in a psycho-social consultation regularly. Psycho-social risk factors were often reported by the women and the psychologist was able to offer support in most cases. Additional support is necessary in some women. Preventive psycho-social support should be considered for women at increased risk of pregnancy-related complications and accessibility needs to be ensured.

## Data Availability

De-identified patient data is available at the online repository OpARA (https://opara.zih.tu-dresden.de).

## Abbreviations

IUGR: Intrauterine growth restriction
PE: Preeclampsia
